# Supplementary material for: Task-induced functional brain connectivity mediates the relationship between striatal D2/3 receptors and working memory
Source: eLife. 2019 Jul 10;8:e45045. doi: 10.7554/eLife.45045 (PMC6620042; doi:10.7554/eLife.45045)
Supplement: Transparent reporting form [file elife-45045-transrepform.pdf]

eLIFE
1st Floor
24 Hills Road
Cambridge CB2 1JP, UK
P 01223 855340
W elifesciences.org
T @elife

eLife's transparent reporting form

We encourage authors to provide detailed information *within their submission* to facilitate the interpretation and replication of experiments. Authors can upload supporting documentation to indicate the use of appropriate reporting guidelines for health-related research (see EQUATOR Network), life science research (see the BioSharing Information Resource), or the ARRIVE guidelines for reporting work involving animal research. Where applicable, authors should refer to any relevant reporting standards documents in this form.

If you have any questions, please consult our Journal Policies and/or contact us: editorial@elifesciences.org.

Sample-size estimation

- You should state whether an appropriate sample size was computed when the study was being designed
- You should state the statistical method of sample size computation and any required assumptions
- If no explicit power analysis was used, you should describe how you decided what sample (replicate) size (number) to use

Please outline where this information can be found within the submission (e.g., sections or figure legends), or explain why this information doesn't apply to your submission:

Our study includes a sample of 48 participants with complete data-sets (both fMRI and PET). This represents one of the largest combined fMRI-PET datasets in the published literature, and the largest single study examining the relationship between dopamine PET measures and functional connectivity fMRI measures.

We powered our study to enable adequate power to detect effects of moderate size ($d > 0.5$).

Our primary fMRI outcome measure was the effect of working memory load on cortical network functional connectivity strength (operationalized as the coefficient of the linear model relating working memory load to network connectivity strength). A sample size of $n=48$ provides 80% power to detect an absolute effect (non-zero coefficient) of $d > 0.40$ (if such an effect exists) at alpha = 0.05 (one-sample t-test, two-tailed).

Our primary PET-fMRI outcome measure was the correlation between striatal D2/3R availability and task-induced change in connectivity strength. A sample size of $n=48$ provides 80% power to detect an absolute effect of rho > 0.38 (if such an effect exists) at alpha = 0.05 (correlation, two-tailed).

Replicates

- You should report how often each experiment was performed
- You should include a definition of biological versus technical replication
- The data obtained should be provided and sufficient information should be provided to indicate the number of independent biological and/or technical replicates
- If you encountered any outliers, you should describe how these were handled
- Criteria for exclusion/inclusion of data should be clearly stated
- High-throughput sequence data should be uploaded before submission, with a private link for reviewers provided (these are available from both GEO and ArrayExpress)

Please outline where this information can be found within the submission (e.g., sections or figure legends), or explain why this information doesn't apply to your submission:

Original sample size and participant exclusions:
The initial study sample consisted of 53 participants, who all completed the n-back fMRI task session. Two participants were excluded from further analysis: one owing to MR imaging artefact caused by orthodontic braces, and a second owing to non-performance of the task ('no response' rates > 3 SD from the group mean). Fifty-one healthy participants were therefore included in the final fMRI analysis (mean age 25.8 years [SD 6.5], 30 male), forty-eight of whom had a [11C]-(+)-4-propyl-9 hydroxy-naphthoxazine ([11C]-(+)-PHNO) PET scan (two participants declined a PET scan, and a third terminated the scan due to nausea).

48 participants in the final sample had complete data-sets (both fMRI and PET). Each participant had only one fMRI and one baseline PET scan (no replicates).

These details are stated clearly in the Methods section.

eLife Sciences Publications, Ltd is a limited liability non-profit non-stock corporation incorporated in the State of Delaware, USA, with company number 5030732, and is registered in the UK with company number FC030576 and branch number BR015634 at the address 1st Floor, 24 Hills Road, Cambridge CB2 1JP | August 2014

Statistical reporting

- Statistical analysis methods should be described and justified
- Raw data should be presented in figures whenever informative to do so (typically when N per group is less than 10)
- For each experiment, you should identify the statistical tests used, exact values of N, definitions of center, methods of multiple test correction, and dispersion and precision measures (e.g., mean, median, SD, SEM, confidence intervals; and, for the major substantive results, a measure of effect size (e.g., Pearson's r, Cohen's d)
- Report exact p-values wherever possible alongside the summary statistics and 95% confidence intervals. These should be reported for all key questions and not only when the p-value is less than 0.05.

Please outline where this information can be found within the submission (e.g., sections or figure legends), or explain why this information doesn't apply to your submission:

Statistical Analysis methods for fMRI and PET analysis are outlined fully in the Methods section.

Statistical analysis was performed using MATLAB (R2017a) and R (version 3.3.1). $P < 0.05$ (two tailed) was considered statistically significant for the primary analyses. We hypothesised that the DMN and TPN would show opposite task-induced connectivity changes with increasing working memory load, and tested this hypothesis using repeated measures ANOVA and post hoc t-tests. Subsequently, we used Spearman's rank correlation coefficient to test for (direct) monotonic relationships between task-induced connectivity changes within cortical networks and both striatal D2/3R availability ([11C]-(+)-PHNO BPND) and task performance (where, for each participant, task-induced connectivity change within a network was defined as the mean w_1 value for all edges in the network). Spearman's rank coefficient was used as we did not assume that the monotonic relationship between variables would be linear. We tested the hypothesis that striatal D2/3R availability influences working memory task performance by modulating cortical network connectivity with a mediation analysis using the 'mediation' package in R, implementing a nonparametric bootstrap method with bias-corrected and accelerated confidence intervals and 10,000 simulation draws. We used the cocor package in R (http://comparingcorrelations.org/) to compare correlation (Spearman's rank) coefficients between (overlapping, dependent correlations) at $P < 0.05$ (2-tailed), using Meng et al.'s method for comparing correlation coefficients (including 95% confidence intervals for difference between correlation coefficients).

(For large datasets, or papers with a very large number of statistical tests, you may upload a single table file with tests, Ns, etc., with reference to sections in the manuscript.)

Group allocation

- Indicate how samples were allocated into experimental groups (in the case of clinical studies, please specify allocation to treatment method); if randomization was used, please also state if restricted randomization was applied
- Indicate if masking was used during group allocation, data collection and/or data analysis

Please outline where this information can be found within the submission (e.g., sections or figure legends), or explain why this information doesn't apply to your submission:

Our study has no group allocation.

Additional data files ("source data")

- We encourage you to upload relevant additional data files, such as numerical data that are represented as a graph in a figure, or as a summary table
- Where provided, these should be in the most useful format, and they can be uploaded as "Source data" files linked to a main figure or table
- Include model definition files including the full list of parameters used
- Include code used for data analysis (e.g., R, MatLab)
- Avoid stating that data files are "available upon request"

Please indicate the figures or tables for which source data files have been provided:

Neuroimaging summary data and analysis scripts (MATLAB and R) will be made publicly available on publication.

eLife Sciences Publications, Ltd is a limited liability non-profit non-stock corporation incorporated in the State of Delaware, USA, with company number 5030732, and is registered in the UK with company number FC030576 and branch number BR015634 at the address 1st Floor, 24 Hills Road, Cambridge CB2 1JP | August 2014
